# Effect of the enzymatically modified supported dipalmitoylphosphatidylcholine (DPPC) bilayers on calcium carbonate formation

**DOI:** 10.1007/s00396-015-3796-0

**Published:** 2015-11-06

**Authors:** Aleksandra Szcześ

**Affiliations:** Department of Physical Chemistry-Interfacial Phenomena, Faculty of Chemistry, Maria Curie-Skłodowska University, Lublin, 20-031 Poland

**Keywords:** Calcium carbonate, Mineralization, DPPC, Phospholipid bilayers, Phospholipase A_2_

## Abstract

After an hour contact with a phospholipase A_2_ (PLA_2_) solution, only the outer leaflet of the dipalmitoylphosphatidylcholine (DPPC) bilayers supported on mica surface underwent hydrolysis whose products, i.e., palmitic acid and lysophospholipid, accumulated on the bilayer surface. Only calcite was present on the bare mica and enzymatically unmodified and modified supported DPPC bilayers soaked for 2 weeks at 25 and 37 °C in a solution of initial pH equals to 7.4 and 9.2 containing calcium and bicarbonate ions at their concentrations about those of human blood plasma. The DPPC bilayers accelerate the crystal growth at lower pH and favors CaCO_3_ nucleation at higher pH. Enzymatic modification of bilayers does not affect crystal morphology and its organization on the examined surface but causes a slight crystal size increase at lower pH and significantly reduces crystal size at alkaline pH. The temperature increase leads to the formation of bigger crystals under physiological pH and has almost no effect on crystal size at alkaline pH. The obtained results are probably attributed to Ca^2+^ interaction with a specific polar site on the surface of the membrane and DPPC hydrolysis products acting as nucleation centers.

## Introduction

Biomineralization, such as bone, tooth, or shell formation, is an important phenomenon in nature. Biominerals possess unique structure and properties, e.g., superior mechanical strength. Much work has been done to understand this process which allows applying biomimetic strategies to produce novel materials for medical, optical, chemical, and electronic applications. Calcium carbonate which is present in corals or shells of marine invertebrates and in gallstones in vertebrates as well as calcium phosphate (main constituent of bones and teeth) are the most abundant biomaterials in nature. Therefore, calcium carbonate is one of the most suitable materials for the research of biomineralization [1, 2]. Calcium carbonate is met as different polymorphs, like (in the increasing solubility order) calcite, aragonite, vaterite, calcium carbonate monohydrate, calcium carbonate hexahydrate, and amorphous calcium carbonate. Calcite is the most thermodynamically stable form. In the biological systems, calcite, aragonite, vaterite, and amorphous calcium carbonate can nucleate and grow stably, and their lattice can be stabilized in the presence of bio-organic macromolecules such as proteins, polysaccharides, and acidic biomacromolecules [3–5].

Biominerals with unique morphologies and hierarchical structures are formed by specific processes involving molecular recognition at organic-inorganic interface [5, 6]. Also, electrostatic attraction, geometrical matching, and stereochemical complementarity may act in the mineralization [6]. There are numerous studies on the effect of different organic matrices to control growth, morphology, structure, and polymorphs of inorganic minerals [5–20].

It is known that cell membranes may also play a role in the biomineralization [21, 22]. Hence, models of biological membranes such as Langmuir and Langmuir-Blodgett films [6, 17, 18] and matrix vesicles (liposomes) [5, 19, 20] have been studied as extracellular matrices to modify the growth of minerals. Cell membranes are mainly composed of phospholipids, and the main component of these phospholipids is choline containing ones [22, 23]. Because phosphatidylcholines are the primary phospholipids in the mammalian cell membrane, dipalmitoylphosphatidylcholine (DPPC) has been frequently a chosen phospholipid in many model studies [6, 16–20].

It is also known that in many physiological processes, the cell hydrolysis of the phospholipids is catalyzed by various phospholipase enzymes. Considering their stereospecificity, phospholipases are specified as A_1_, A_2_, C, and D [24, 25]. Phospholipase A_2_ (PLA_2_) is involved in diverse pathologies in humans, including inflammation, tissue repair, and cancer. Therefore, they have attracted great attention. Phospholipase A_2_ catalyzes the hydrolysis of phospholipids at the sn-2 position producing free fatty acids and lysophospholipids. The presence of hydrolysis products in the membrane modulate its composition and can alter its physicochemical properties [25–27]. Hence, studies on mineralization of enzymatically modified solid supported phospholipids layers may be useful either in the determination of factors controlling biomineralization or potential biomedical applications (e.g., in implantology). Bone repair is a process of reconstruction of bone tissue in the area of injury. However, because of the extent of bone loss or damage, the bone self-repair mechanism is sometimes insufficient for full regeneration. This leads to search for artificial substitutes for bone grafts which unfortunately may cause immunological response when implanted. Hence, a biocompatible implant surface is needed. Recently, phospholipid multilayers are investigated as materials leading to the improvement of the contact between cells and the implant surface [28]. Thus, the present study may provide information on how changes in the phospholipid layers caused by phospholipase A_2_ present in chronic inflammation or infection may alter bone formation and mineralization.

In the previous study concerning phosphate mineral formation on the supported DPPC mono- and bilayers, it was shown that the DPPC bilayers enhance formation of less soluble phosphate forms, especially at physiological temperature equal to 37 °C [16]. The aim of the current study is to evaluate whether enzymatic modification of DPPC bilayers has an effect on calcium carbonate mineralization. Because of the participation of phospholipase A_2_ in the inflammatory processes, this study could provide information about pathological membrane mineralization and may be useful either in the determination of factors controlling biomineralization or potential biomedical applications (e.g., in implantology).

## Experimental

### Materials

1,2-Dipalmitoyl-*sn*-glycero-3-phosphocholine (DPPC; semi-synthetic, 99 %) and PLA_2_ (lyophilized powder from *bovine pancreas*, specific activity ≥20 units/mg) were supplied by Sigma and were used without further purification. Sodium chloride, calcium chloride, sodium bicarbonate, and Tris (tris-(hydroxymethyl)aminomethane) were of p.a. grade from Avantor Performance Materials Poland S.A., Poland. DPPC was dissolved in a chloroform (p.a., Avantor Performance Materials Poland S.A.) to obtain a concentration equal to 1 mg/ml. The water used was purified by a Milli-Q plus water purification system (Millipore, USA) with a resistivity of 18.2 MΩ cm.

PLA_2_ as a powder was dissolved in Tris buffer solution (5 mM CaCl_2_, 10 mM NaCl (POCH S.A., Poland) and 10 mM tris(hydroxymethyl)aminomethane) of pH = 8.1. The enzyme concentration was equal to 0.002 units/ml.

### DPPC layer formation and characterization

The Langmuir-Blodgett/Langmuir-Schaefer (LB/LS) technique was used to prepare the lipid bilayers supported on freshly cleaved mica (muscovite, Continental Trade, Warsaw). The preparation of the DPPC layers was carried out using a commercial Langmuir-Blodgett trough (KSV 2000, Finland) and followed the procedure described by Jurak et al. [27]. The DPPC monolayer was transferred vertically onto the mica support at a surface pressure of 35 mN/m and 5 mm/min rate. After 15 min of waiting between the first and second layer deposition, the bilayer was deposited via the LS method by horizontal touching of DPPC monolayer on the mica to previously deposited monolayer on the water subphase in the LB trough. These processes were carried out at 20 °C. Immediately after transferring onto the solid support, the bilayers were rapidly dipped into the “mineralizing solution” of initial pH 7.4 and 9.2 containing 2.6 mM Ca^2+^ and 4.2 mM HCO_3_^−^ ions at a constant temperature of 25 or 37 °C. At alkaline pH, the properties of hydrolysis products (free fatty acid and 1-acyl-lyso-phospholipid), i.e., their solubility and charge, differ from those at pH about 7. In particular, ionized fatty acid may promote calcium ions binding through electrostatic interaction. Hence, apart from experiments conducted at physiological pH, also, experiments at alkaline pH = 9.2 were performed to learn whether it can influence the lipid membrane mineralization. Concentration of calcium and bicarbonate ions corresponded to the simulated body fluid (SBF) in which the ion concentrations are nearly equal to those of human blood plasma [29].

The enzymatically modified DPPC bilayer was obtained by immersing the bilayer-coated mica slides in the PLA_2_ solution for 1 h at 25 °C, just after the phospholipid deposition. After that, the mica slide was rinsed three times with distilled water and immersed in the solution for mineralization.

The surface topography of freshly cleaved mica and phospholipid bilayers was examined using the atomic force microscopy (AFM) (NanoScope V, Bruker-Veeco, USA) with a help of WSxM 5.0, Develop 6.4 Scanning Probe Microscope software [30]. For this purpose, when the enzyme exposition time was completed, the samples were dipped three times in Milli-Q water and dried in vacuum desiccators (Binder with a pump CVC 2000) under the pressure of 117 mbar for about 24 h. The bilayer-coated mica slides were also dried in vacuum desiccator for 24 h before AFM measurement.

Such prepared phospholipid bilayer surface without and after PLA_2_ modification was also characterized by X-ray photoelectron spectroscopy (XPS). XPS analyses were performed with UHV Prevac spectrometer with a monochromatized aluminum X-ray source (30 mA, 12 kV) and a charge stabilization devices. The binding energy was set by fixing the component of the C1s peak at 284.8 eV. Obtained spectra were decomposed using CasaXPS software.

### Mineral phase deposition and characterization

Mineralization process was conducted as described in the earlier published paper [16]. Briefly, the freshly cleaved bare mica and DPPC-coated mica slides, whose size was about 3.5 cm × 1.5 cm, were immersed vertically in the solution for 14 days to avoid as much as possible deposition of the precipitated calcium carbonate in the bulk solution which would next sediment on the slide surface. The 30 ml of mineralizing solutions were placed in plastic boxes at 25 and 37 °C. During that 14-day time, the solution was changed every 3 days by its fresh portion. Then, the mica slides were removed, rinsed with distilled water, and dried in a desiccator at room temperature.

The crystal forms and morphology of the precipitated mineral phase were determined by Raman spectra (inVia Reflex instrument, Renishaw, UK) and scanning electron microscopy (Quanta 3D FEG, FEI).

## Results

### DPPC layer characterization

**Fig. 1:**
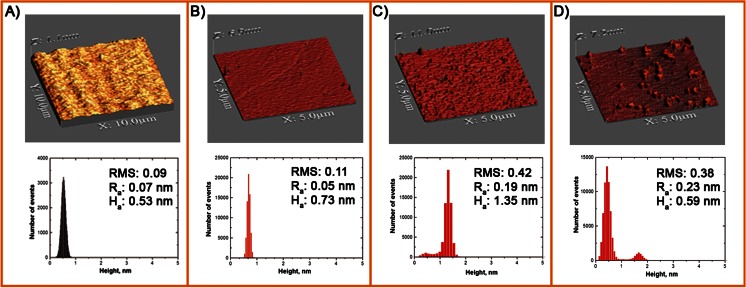
AFM 3D images and height distribution determined for **a** the bare mica and DPPC bilayer surface on mica: **b** dried, **c** after 60 min exposition to Tris buffer solution (pH = 8.1), and **d** after 60 min exposition to PLA_2_ solution (0.002 units/ml, pH 8.1)

Atomic force microscopy (AFM) technique provides information on the structure and properties of the supported lipid layers. This technique was applied to characterize the surface of the phospholipid supported bilayer before and after the phospholipase A_2_ modification. Figure 1 shows 10 μm × 10 μm AFM images presenting topography of the bare mica and 5 μm × 5 μm AFM images of DPPC bilayer before and after the hydrolysis.

The corresponding topography parameters (root mean square (RMS), roughness average (*R*_a_), height average (*H*_a_)) are placed in this figure. As can be seen in Fig. 1a, the surface of bare mica is smooth which is confirmed by small topography parameters. The roughness of DPPC bilayer not contacted with water (Fig. 1b) is also relatively small (RMS = 0.11, *R*_a_ = 0.05 nm). The distribution of the protrusions is narrow, and their heights are mostly between 0.5 and 1 nm with maximum intensity around 0.75 nm. It proves that the DPPC bilayer supported on mica surface is smooth and homogenous. After 60 min exposition to Tris buffer solution, some peaks and valleys can be observed (Fig. 1c). The average roughness increases four times to 0.19 nm. The distribution of protrusions is broader with their maximum intensity around 1.5 nm. This indicates that the bilayer structure after its contact with the solution becomes less uniform than that uncontacted. However, as can be seen in Fig. 1d, after 60 min contact with the enzyme, the film structure is dramatically changed where small islands spread over the surface appear. In comparison to the bilayer contacted with the solution without the enzyme, although the roughness average is almost the same, the average height decreases twice, from 1.35 to 0.59 nm. Similarly, distribution of the protrusions lies within the same range, i.e., between 0 and 2 nm, but their maximum intensity is shifted toward lower value, e.g., around 0.5 nm. This means that the surface becomes more inhomogeneous as a result of accumulation of the hydrolysis products (palmitic acid and 1-palmitoyl-2-hydroxy-sn-glycero-3-phosphocholine). Basing on the height distribution, one may claim that the hydrolysis process initiates in the outer leaflet of the bilayer, since the thickness resulting from the distribution of the protrusions (up to 2 nm) corresponds with the monolayer and not the bilayer thickness [30, 31] which is consistent with the results obtained by Tong et al. [32]. Besides, the high difference of ca. 1.5 nm (Fig. 1d) can be associated with the presence of the hydrolysis products [33]. Hence, DPPC bilayers become a mixture of DPPC, palmitic acid, and lyso-palmityoyl-PC.

It is known that the PLA_2_s are water soluble and act when they adsorb at a membrane surface, and the rate of hydrolysis depends among others on the way the substrate molecules are organized, i.e., the initial structural defects in the bilayer as well as the compositional defects [32–35]. Vacklin et al. [34] found that at 25 °C initially, phospholipase A_2_ causes the destruction of the bilayer and then the extent of DPPC bilayer destruction is dramatically reduced. Because in the case of DPPC molecule, both hydrolysis reaction products possess saturated chains and they are less likely to leave the lipid bilayers. Hence, aggregation of DPPC reaction products and their accumulation in the bilayer may serve as inhibiting effect. It is proposed that the fatty acid increases the packing density of DPPC and the enzyme cannot access the bonds to be hydrolyzed. They also found that after ca. 14 % of the DPPC bilayers have left the interface, hydrolysis stopped. Similar slowing in the hydrolysis rate was observed by Grandbois and co-workers [33].

In this study, kinetics of bilayer hydrolysis which is reflected in the growth rate of the DPPC bilayer defects was not determined. Nevertheless, as was reported by Chibowski et al. [36] at room temperature, DPPC supported bilayer is degraded by PLA_2_ enzyme within 5–10 min, even if small changes in the surface energy occur even up to 2 h.

XPS analyses were used to obtain complementary information to AFM on the surface chemical composition and bonding of the supported DPPC bilayers before and after the enzyme action. Table 1 shows the relative atomic percentage in examined samples.

**Table 1:** Atom percentage as analyzed by XPS

Sample	C1s (%)	O1s (%)	N1s (%)	P1s (%)	Si2p (%)	P/C
Mica	1.3	51.5	0.0	0.0	23.2	0.000
DPPC bilayers on mica	31.1	35.9	1.1	0.8	16.2	0.026
Enzymatically modified DPPC bilayers on mica	26.8	38.2	1.1	0.9	15.6	0.033

The bare mica surface contains 23.2 % of Si and no nitrogen and phosphorous (Table 1). After DPPC bilayer deposition on its surface, the silicon content is reduced to 16.2 atom percentage, and nitrogen and phosphorous appear confirming that the phospholipid is grafted to the mica substrate. It can be also seen that the N/P ratio for enzymatically untreated and treated supported bilayers is close to the theoretical value of 1:1. After PLA_2_ action, it can be seen that the C1s content decrease from 31.1 % of unmodified DPPC bilayer to 26.8 % for enzymatically modified DPPC bilayers. Almost constant content of N1s and P1s and the P/C ratio increase may be the result of palmitic acid release during the enzyme action and 1-palmitoyl-2-hydroxy-sn-glycero-3-phosphocholine accumulation on the surface.

Because the decrease of carbon content does not correspond to the loss of one palmitic acid chain from one DPPC molecule, it probably also accumulates on the surface.

**Fig. 2:**
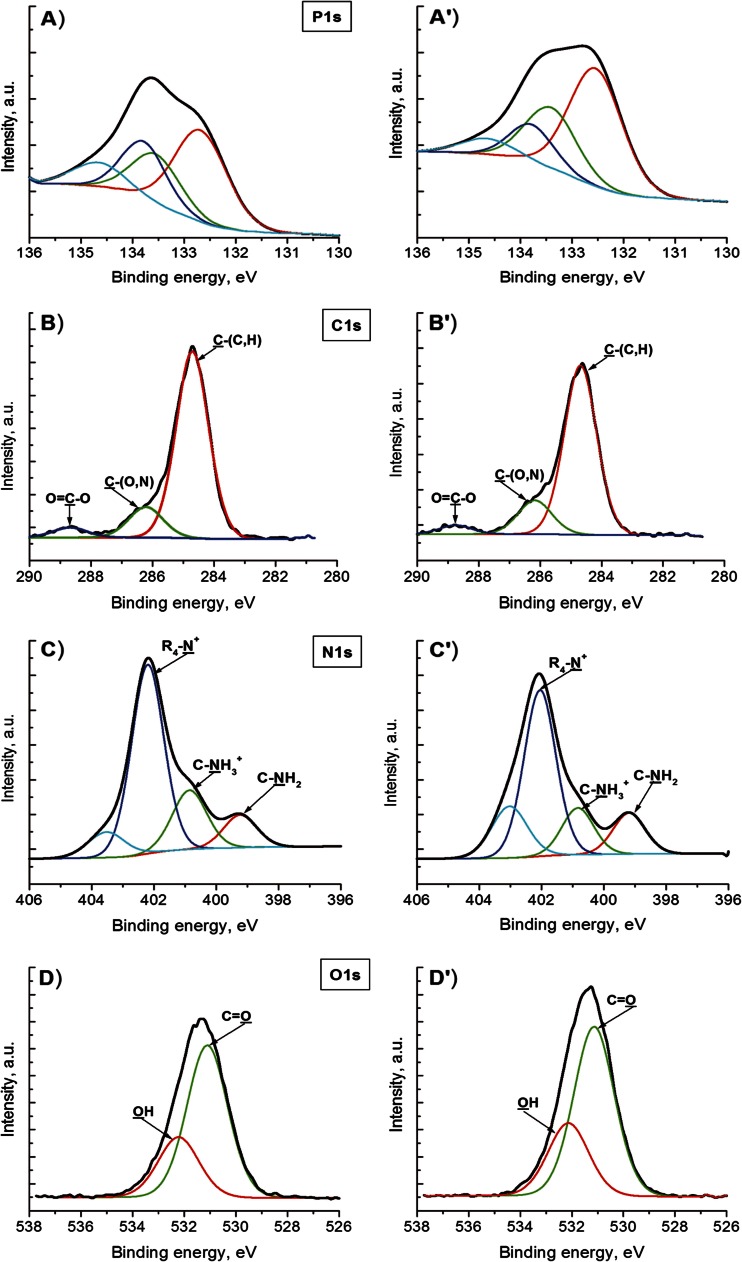
XPS peaks (P1s, C1s, N1s, and O1s) recorded on DPPC layers deposited on mica surface: *A*–*D* enzymatically unmodified and *A*’–*D*’ after modification with PLA_2_

Narrow scan spectra and curve fitting for the phospholipid bilayers before and after the enzyme action are shown in Fig. 2.

It can be seen that similar results were obtained for DPPC supported bilayers without and after PLA_2_ action. A distinct peak for phosphorous at ca. 134 eV confirms the presence of the phosphorous containing bond. The C1s peak is decomposed to three peaks at 284.7, 286.2, and 288.7 eV with attributions to C–(C,H), C–(O,N), and O=C–O functions. The component at 402.2 eV is attributed to quaternary ammonium (R_4_–N^+^) of the phospholipid headgroup. The oxygen peak (O1s) is decomposed in two peaks at 531.1 and 532.2 eV corresponding respectively to oxygen doubly bound to carbon due to carboxyl and C–OH of alcohol [37, 38].

The existence of peak for phosphorous (133.3 eV) and for quaternary nitrogen (402.2 eV) provides an evidence that the phospholipid is grafted to the mica substrate.

### Characterization of mineral deposited at initial pH = 7.4

**Fig. 3:**
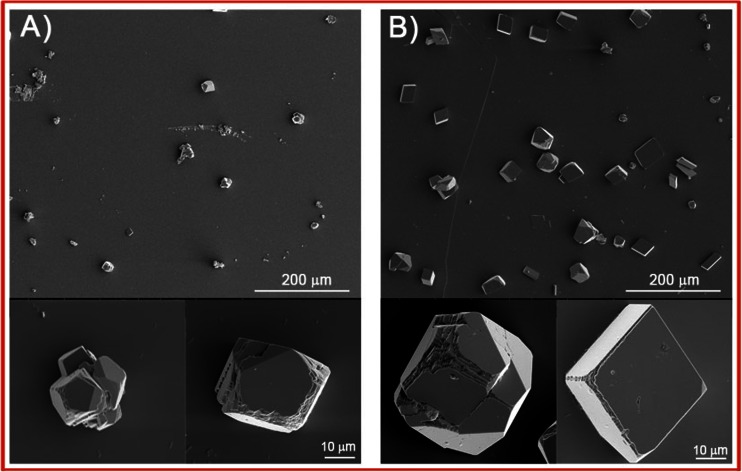
Representative SEM images of calcium carbonate microcrystals deposited at initial pH = 7.4 on bare mica slides at **a** 25 °C and **b** 37 °C

**Fig. 4:**
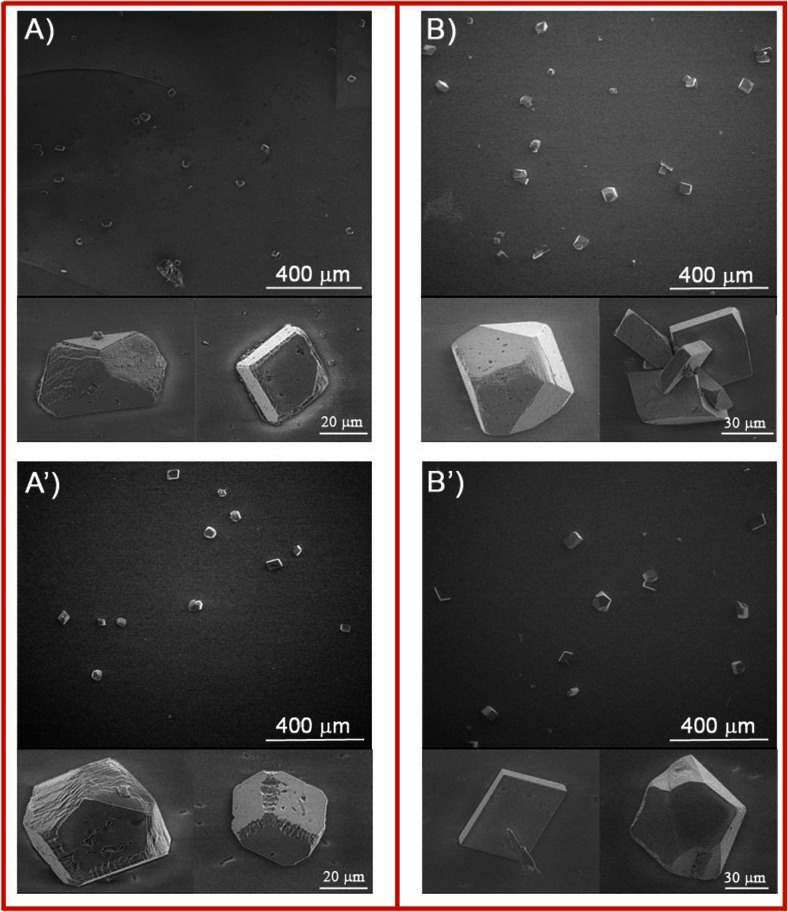
Representative SEM images of calcium carbonate microcrystals deposited at initial pH = 7.4 at 25 °C (*A*, *A*’) and 37 °C (*B*, *B*’) on DPPC bilayer-coated mica slides: *A* and *B* enzymatically unmodified, and *A*’ and *B*’ PLA_2_ modified

Next, the bare mica slides and DPPC bilayer-covered mica slides after the phospholipid deposition were immediately immersed into the mineralizing solution for 14 days. To investigate DPPC bilayer modification by phospholipase A_2_ on the deposition of calcium carbonate, before the slide immersion in the “mineralization solution,” the lipid bilayers were soaked for 1 h in the enzyme solution. The precipitated crystals were characterized by SEM and Raman spectroscopy. Representative SEM images of the surfaces mineralized at initial pH = 7.4 are shown in Figs. 3 and 4. The insets show crystals in higher magnification.

**Fig. 5:**
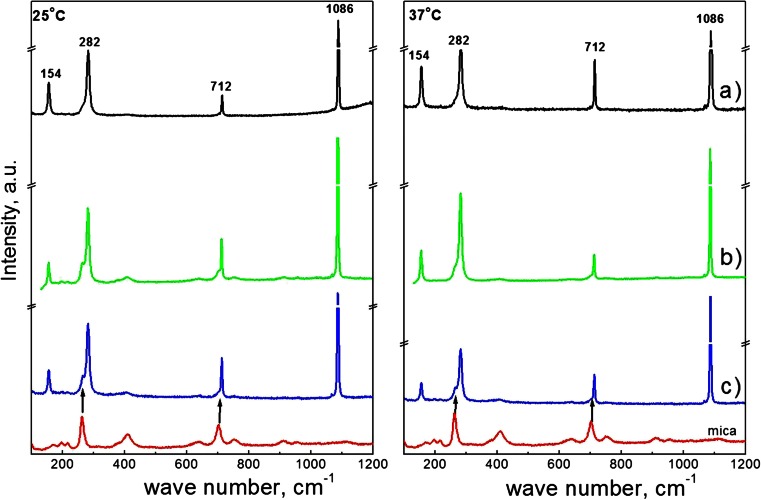
Raman spectra of slides of bare mica and with calcium carbonate deposited at 25 and 37 °C at initial pH = 7.4 on *a* bare mica, *b* DPPC bilayers on mica, and *c* DPPC bilayers after contact with the enzyme

When the mineral was deposited on bare mica surface at 25 °C, obtained crystals are of distorted morphology from rhombohedral to spherical with rough edges (Fig. 3a). At higher temperature, more crystals of rhombohedral and distorted morphology appear (Fig. 3b). Moreover, obtained particles become bigger, indicating that temperature increase favors precipitation of larger crystals. From Fig. 4, it can be seen that at both investigated temperatures on enzymatically modified and unmodified DPPC bilayer surface, the crystals of plate-like morphology and distorted morphology, from rhombohedral to spherical shapes, are obtained. It is worth noticing that the crystals have a terraced structure in the corners and small cavity on their surfaces. This may indicate that small crystals underwent dissolution and larger were formed. Moreover, enzymatic modification of DPPC bilayers does not change morphology of the obtained crystals. If mineralization was carried out on the DPPC bilayers at 25 °C, the crystals with size 30–45 μm are distributed on the surface (Fig. 4(A)). As a consequence of the temperature increase, the crystals of the bigger size range from 45 to 75 μm are formed (Fig. 4(b)). These crystals’ size is significantly bigger in comparison to crystals obtained on bare mica surface. It shows that the presence of the phospholipid bilayers makes the size of the crystals larger. At both investigated temperatures, calcium carbonate particles obtained on the enzymatically modified DPPC bilayers exhibit the same morphology like those obtained on unmodified phospholipid bilayers; however, they are slightly bigger. The corresponding Raman spectra of calcium carbonate precipitated at 25 and 37 °C on the bare mica, as well as on phospholipid bilayers without and after the enzyme modification, are shown in Fig. 5.

All of these spectra show bands at 154, 282, 712, and 1086 cm^−1^ indicating the existence of calcite [39]. The small peak at 264 cm^−1^ and about 701 cm^−1^ for the samples obtained on DPPC bilayer without and with the enzyme action at 25 °C, and on enzymatically modified DPPC bilayer at 37 °C, does not indicate coexistence of vaterite and aragonite but most probably can be assigned to mica which exhibits the strongest absorption bands at this wave numbers. From the Raman spectra, it can be concluded that thermodynamically, stable calcite is the only polymorph precipitated on the bare mica and the supported DPPC bilayers. Enzymatic modification of the phospholipid layers does not change the obtained calcium carbonate crystal form.

### Characterization of mineral deposited at initial pH = 9.2

**Fig. 6:**
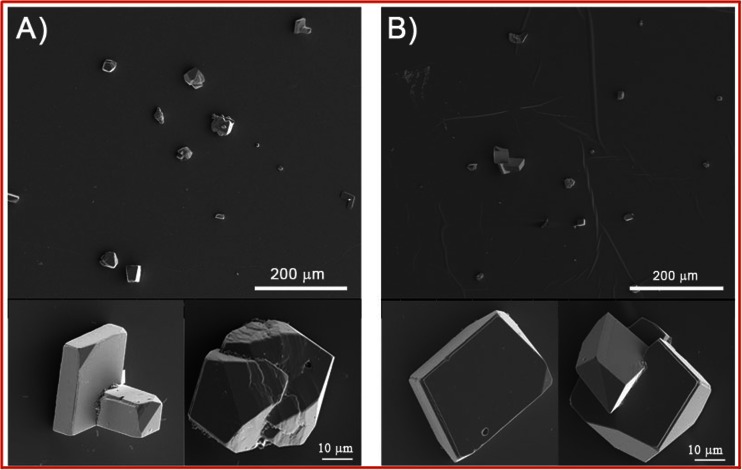
Representative SEM images of calcium carbonate microcrystals deposited at initial pH = 9.2 on bare mica slides at **a** 25 °C and **b** 37 °C

Figure 6 shows crystals deposited on bare mica at 25 and 37 °C at initial pH equals to 9.2. Compared with the results at physiological pH = 7.4, the number of crystals on the mica surface decreases, to a large extent at higher temperature. At 25 °C, a bigger crystal of similar morphology as obtained at lower pH can be seen. In contrast to the results presented in Fig. 4, the temperature increase does not cause crystal size increase. Among a few big crystals, smaller ones with smoother edges appear.

**Fig. 7:**
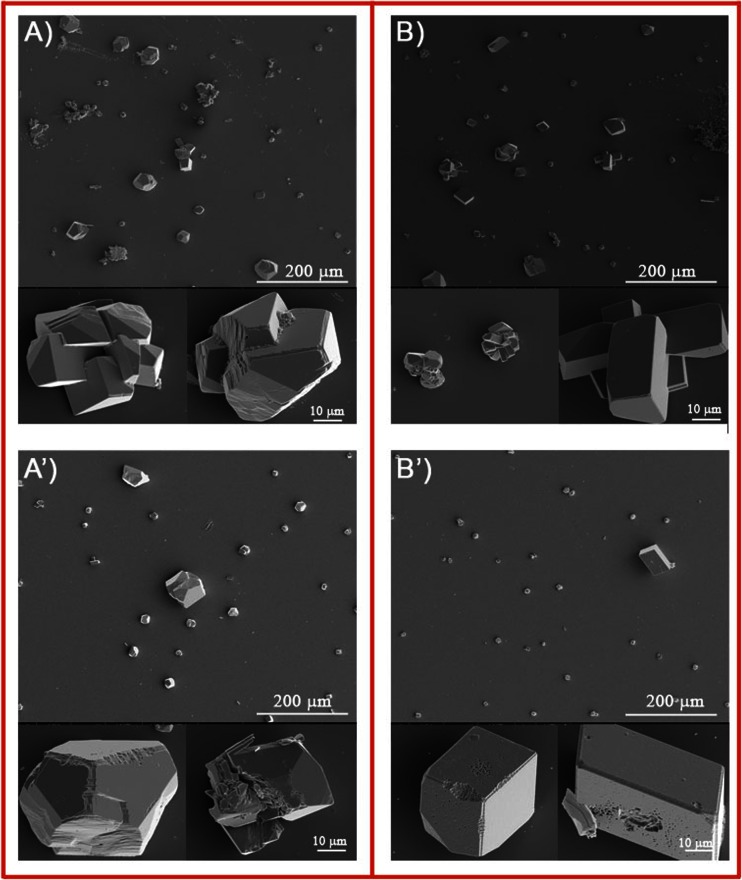
Representative SEM images of calcium carbonate microcrystals deposited at initial pH = 9.2 at 25 °C (*A*, *A*’) and 37 °C (*B*, *B*’) on DPPC bilayer-coated mica slides *A* and *B* enzymatically unmodified and *A*’ and *B*’ PLA_2_ modified

The growth of CaCO_3_ on DPPC bilayers at higher initial pH leads to formation of aggregates consisting of smaller distorted rhombohedral crystals of rough edges (Fig. 7). Moreover, more crystals are deposited on the supported phospholipid surface. At both investigated temperatures (Fig. 7(A, B)), mineral deposit contains mixture of big and much smaller aggregates. However, at 37 °C, crystals of smooth edges and well-defined surface are formed. Single crystals with distorted morphology are present on enzymatically modified DPPC bilayers at 25 °C and rhombohedral crystals with well-defined surface and edges at 37 °C (Fig. 7(A’, B’), respectively). At both investigated temperatures, enzymatic modification of bilayers leads to formation of smaller crystals than on the unmodified phospholipid bilayers. As in the case of mineral deposited on bare mica surface, temperature increase does not cause crystal size increase.

**Fig. 8:**
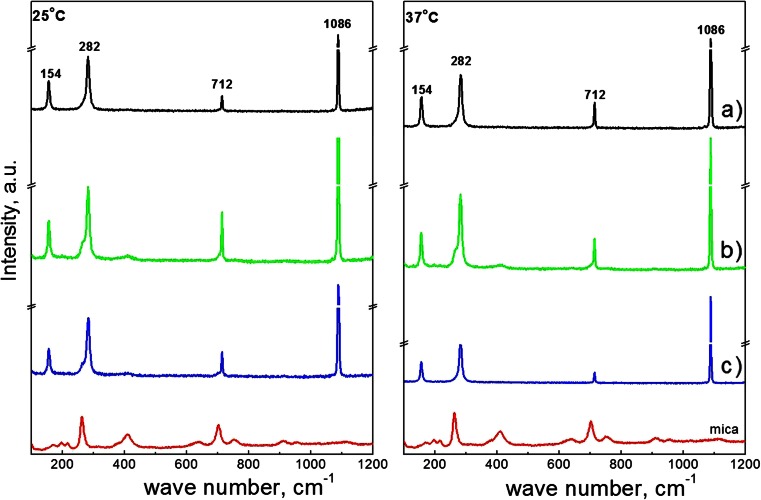
Raman spectra of slides of bare mica and with calcium carbonate deposited at 25 and 37 °C at initial pH = 9.2 on *a* bare mica, *b* DPPC bilayers on mica, and *c* DPPC bilayers after contact with the enzyme

Raman spectra corresponding to crystals presented on Figs. 6 and 7 are shown in Fig. 8. In a similar way as under physiological pH only, bands characteristic for calcite appear. It can be seen that pH does not influence the polymorph of calcium carbonate precipitated on mica surface and on enzymatically unmodified and modified DPPC bilayers but influence its shape, morphology, crystal organization, and crystals size. Surprisingly at higher initial pH, temperature increase has almost no effect on the size of crystals obtained on bare mica as well as on DPPC-covered mica surface.

## Discussion

Generally, a mineral formation involves two major steps: nucleation and growth of crystals, which in the case of biomineralization occur within organic matrices [5, 6, 40]. The polymorph, size, and shape of the crystals are recognized to be controlled by the size, shape, and surface charge density of the organic matrix which acts as a template for the mineral microstructure [5, 6, 41]. Nucleation may be initiated by the adsorption of cations onto functional sites of acidic macromolecules, which promotes the formation of critical nuclei [5, 6, 19]. Crystallite orientation may be controlled by specific molecular interactions, which causes the arrangement of solution ions in specific locations relative to organic sites [40]. It is known that ions interact with charged phospholipids via Coulombic forces and the apparent association of metal cations with lipid membranes is distinctly more intense for anionic lipids than for neutral, zwitterionic ones. However, the exposure of phosphate groups affects the interaction of calcium ions with the lipid membrane [42].

**Fig. 9:**
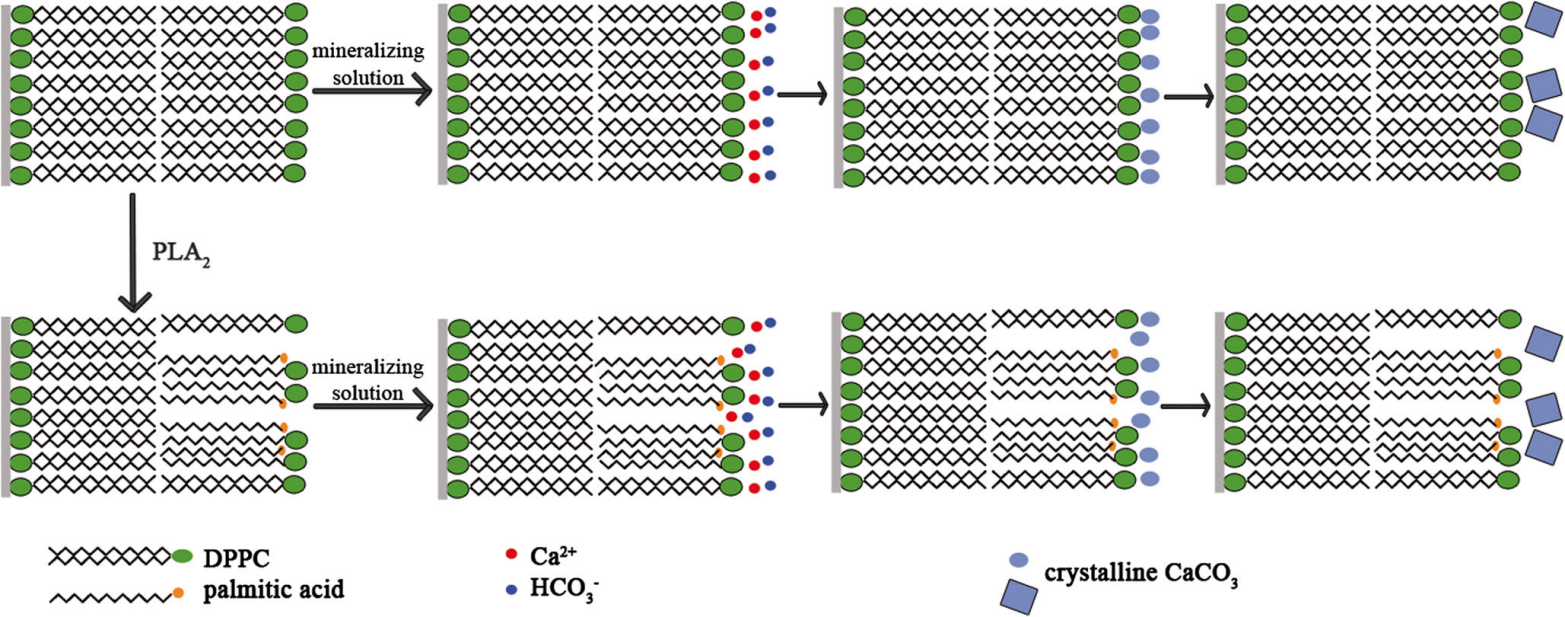
Proposed mechanism of calcium carbonate formation on supported DPPC bilayers

The polar part of phospholipid molecules contains at least one (usually several) ionizable group. The electrical charge of DPPC molecule is concentrated in the hydrophilic zwitterionic headgroup of the phospholipids which possesses two spatially separated and oppositely charged moieties: positive choline group –N^(+)^(CH_3_)_3_ and negatively charged—*OPO*_3_—group [43]. Hence, the headgroup of DPPC molecules may attract calcium ions forming complex of the phosphate group with calcium ions [6, 19, 34] acting as nucleation centers. This leads to the enrichment of Ca^2+^ near the membrane surface, which sequentially attract more bicarbonate ions giving more calcium carbonate crystals than on bare mica surface. As was found from AFM images and XPS analysis, the hydrolysis process is initiated in the outer leaflet and the hydrolysis products (1-palmitoyl-2-hydroxy-sn-glycero-3-phosphocholine and palmitic acid) accumulate on the surface. As a result of the hydrolysis, one DPPC molecule gives two molecules which can interact with the calcium ions. Moreover, the release of one acid molecule from the phospholipid causes higher affinity of the phosphate groups toward the aqueous phase and hence better exposure of these groups to interact with calcium ions (Fig. 9). Consequently, the number of nucleation centers is not reduced significantly. In addition, an increase in pH may enhance binding of metal ions to acidic moieties such as the phosphate group or carboxyl group of long-chain acid released by hydrolysis.

The increase in Ca^2+^ saturation in the vicinity of the lipid surface favors nucleation and precipitation according to the Gibbs-Thomson formula of classical nucleation theory [12]. More nuclei present on the surface may cause higher local concentration of calcium and bicarbonate ions that appeared during dissolution of the precursor phase and its transformation to less soluble one, i.e., calcite. However, further experiment should be conducted to confirm formation of more soluble precursor phases of calcium carbonate (amorphous calcium carbonate and vaterite) and their transformation to less soluble calcite via dissolution and reprecipitation process.

## Conclusions

The mineralization of calcium carbonate on bare mica surface and on the supported enzymatically unmodified and modified DPPC bilayers was investigated. One hour contact with PLA_2_ solution resulted in changes in the outer leaflet of the bilayer and accumulation of the hydrolysis products on the surface. It was found that after 2 weeks immersion in the solution, calcite was the only form present on all investigated surface, i.e., bare mica and enzymatically unmodified and modified DPPC bilayers. The presence of the phospholipid bilayers accelerate the crystal growth at initial pH equals to 7.4 but favors nucleation in the system of more alkaline initial pH (more crystals forming aggregates appeared).

It was found that the phospholipid bilayer modification with the phospholipase A_2_ does not affect crystal morphology and its organization on the surface but causes slight crystal size increase at lower pH and significantly reduces crystals size at more alkaline pH. This is probably due to the fact that as a result of hydrolysis, one DPPC molecule produces two molecules interacting with calcium ions and causes better exposure of the phosphate group to interact with calcium ions. Moreover, pH increase from 7.4 to 9.2 enhances ionization of the acidic lipid component such as the carboxyl group of long-chain acid released by hydrolysis which leads to the enhancement of calcium ion binding. Under physiological pH, the temperature increase from 25 to 37 °C enhances formation of crystals of a bigger size, while at alkaline pH = 9.2, temperature increase has almost no effect on size of crystals obtained on bare mica as well as on DPPC-covered mica surface.
